# Predictors of Early Neurological Deterioration in Stroke Due to Vertebrobasilar Occlusion

**DOI:** 10.3389/fneur.2021.696042

**Published:** 2021-09-14

**Authors:** Seungyon Koh, Sung Eun Lee, Woo Sang Jung, Jin Wook Choi, Jin Soo Lee, Ji Man Hong, Seong-Joon Lee

**Affiliations:** ^1^Department of Neurology, Ajou University School of Medicine, Ajou University Medical Center, Suwon, South Korea; ^2^Department of Emergency Medicine, Ajou University School of Medicine, Ajou University Medical Center, Suwon, South Korea; ^3^Department of Radiology, Ajou University School of Medicine, Ajou University Medical Center, Suwon, South Korea

**Keywords:** vertebrobasilar artery occlusion, early neurological deterioration, endovascular treatment, mechanical thrombectomy, posterior circulation stroke

## Abstract

**Background and Aims:** This study explores the predictors of early neurological deterioration (END) in patients with vertebrobasilar occlusion (VBO) in both primary endovascular therapy (EVT) and medical management (MM) groups.

**Methods:** Patients diagnosed with VBO from 2010 to 2018 were included. Comparative and multivariate analyses were used to identify predictors of all-cause END in the EVT group, and END due to ischemia progression (END-IP) in the MM group.

**Results:** In 174 patients with VBO, 43 had END. In the primary EVT group (*N* = 66), 17 all-cause END occurred. Distal basilar occlusion (odds ratio (OR), 14.5 [95% confidence interval (CI), 1.4–154.4]) and reperfusion failure (eTICI < 2b67 (OR, 5.0 [95% CI, 1.3–19.9]) were predictive of END in multivariable analysis. In the MM group (N=108), 17 END-IP occurred. Higher systolic blood pressure (SBP) at presentation (per 10 mmHg increase, OR, 1.5 [95% CI, 1.1–2.0]), stroke onset-to-door time <24 h (OR, 5.3 [95% CI, 1.1–2.0]), near-total occlusions (OR, 4.9 [95% CI, 1.2–19.6]), lower posterior circulation-Alberta Stroke Program Early CT scores (OR, 1.6 [95% CI, 1.0–2.5]), and lower BATMAN collateral scores (OR, 1.6 [95% CI, 1.1–2.2]) were predictive of END-IP.

**Conclusions:** In patients with stroke due to VBO, potential predictors of END can be identified. In the primary EVT group, failure to achieve reperfusion and distal basilar occlusion were associated with all-cause END. In the MM group, higher SBP at presentation, onset-to-door time less than 24 h, incomplete occlusions, larger infarct cores, and poorer collaterals were associated with END-IP.

## Introduction

Early neurological deterioration (END) occurs in up to one-third of patients with acute ischemic stroke and considerably affects outcome (1). With the advent of the stroke endovascular treatment (EVT) era, END is regaining focus for a few reasons. First, prediction of individual patient risk of END can identify candidates that can benefit from EVT, especially those with a lower National Institutes of Health Stroke Scale (NIHSS) score (2). Second, even with reperfusion therapy, END may occur, both associated with incomplete reperfusion (3) and even after successful reperfusion (4); namely unexplained END. However, the aforementioned concerns regarding END have not been appropriately addressed in vertebrobasilar occlusion (VBO).

Identification of factors predictive of END in VBO patients treated with EVT is important in VBO for a few reasons. First, the brain tissue of posterior circulation is more eloquent, and the consequences of incomplete reperfusion can be more critical. Second, the collateral vasculature of the posterior circulation may be more likely to be effected by procedural complications, as the primary Willisian collateral is distally located in the vascular bed (basilar top) in contrast to the proximal location of the anterior circulation. Careful identification of END predictive factors is needed to maximize EVT treatment effect in VBO.

Identification of factors predictive of END in VBO patients with medical management may provide keys to expand the pool of patients that may benefit from EVT. Currently, there is no successful clinical trial that shows the superiority of EVT for VBO (5), but it is likely that through reasonable patient selection, EVT for VBO will result in good outcomes (6). However, patient selection by strict NIHSS scores and time parameters is more difficult to apply in VBO for a few reasons. First, the clinical severity of posterior circulation stroke may be underrated by NIHSS scores (7). Second, the frequency of atherosclerotic disease involving the vertebrobasilar artery is higher, resulting in fluctuating prodromal symptoms, unclear stroke onset, and neurological deterioration (8). Furthermore, in clinical practice, it may be important to identify more patients that may benefit from EVT, rather than to over-select (9). Patients that experience END during medical treatment may be potential candidates if their cause of END is ischemia progression (10) in specific.

As stated above, the general EVT criteria are guided by clinical severity, time metrics, identification of large vessel occlusion, and infarct volume. However, in clinical practice, the decision should be, and tends to be, individualized (9). There may be specific predilections stroke neurologists already have in this selection process in concern of END. In other words, their decision to perform or not to perform EVT may already be affected by their belief that some clinical and imaging findings may be associated with END.

Therefore, through a single-center registry, our goal was to explore potential predictors of END in patients with VBO. In the primary EVT population, we aimed to identify the clinical significance and predictors of all-cause END. In the medical management (MM) population, we identified the clinical significance and predictors of END specifically due to ischemia progression (END-IP). Finally, to clarify the predilections the clinicians already have in the selection of patients for EVT in VBO, we evaluated patients that deviated from the institutional EVT selection criteria.

## Materials and Methods

### Patient Selection

From a university hospital ischemic stroke registry, patients with posterior circulation stroke were identified between 2010 and 2018 (*N* = 1,710). In this cohort, medical record search and analysis of baseline non-invasive angiography were performed to identify patients presenting with occlusion or near-total occlusion in the basilar artery (BA), bilateral vertebral arteries (VA), or dominant VA with contralateral flow absence. Patients in whom VBO occurred during hospitalization for ischemic stroke in another vascular territory were excluded.

The patients were classified into the primary EVT group if EVT decision was made at presentation in the emergency department by the attending stroke neurologist based on the institutional indication of EVT for VBO. The institutional EVT indications were as follows: occlusion of the basilar artery, bilateral V4, or unilateral V4 with contralateral hypoplasia, NIHSS ≥ 5 (if NIHSS < 5, when clinical progression is highly expected), and onset-to-puncture ≤ 6 h (if 6–24 h, decision based on advanced imaging). In specific, EVT performed on the late time window (onset-to-puncture between 6 and 24 h) was also included in the primary EVT group if EVT decision was made at presentation to the emergency department. Otherwise, all patients who did not undergo EVT at presentation were classified into the MM group, in which the patients received standard antithrombotic treatments. Intravenous (IV) thrombolysis was performed when indicated, and IV thrombolysis did not affect patient grouping.

EVT was performed as a “rescue” therapy in some patients after the occurrence of END during the admission course. The decisions on the rescue EVT were made regardless of the initial intent for the treatment of the patients and did not affect patient grouping.

All EVT patients in this population were primarily treated with stent retriever thrombectomy or direct aspiration thrombectomy. The Ajou University Hospital Institutional Review Board approved this research (MED-MDB-20-268), and the board waived the need for patient consent.

### Clinical Variables and Classification of END

The clinical severity was measured by serial NIHSS scores which were evaluated upon first evaluation by the neurologist, and routinely per 6 h after admission to stroke unit by professional stroke nurses. Presenting NIHSS scores were further trichotomized to ≤5, 6–20, and >20. Functional outcomes were measured by the modified Rankin scale (mRS) at 3 months.

END was defined as an increase in the NIHSS score compared with the best neurological status by more than four points during admission (1). The causes of END were separately classified in the primary EVT group and MM group.

In the primary EVT group, END caused by immediate and overt subarachnoid or intraventricular hemorrhages post-procedures were first classified as procedural complications. Next, reperfusion was graded based on the expanded treatment in cerebral ischemia (eTICI) scores (11), and successful reperfusion was classified as ≥eTICI2b67 (reperfusion of more than two-thirds of involved territory). In patients with reperfusion failure, all ENDs were considered as the result of reperfusion failure. In patients with successful reperfusion, causes of END were classified as cerebral edema, symptomatic hemorrhage, or reocclusion. Patients with END due to unexpected, severe medical problems disproportionate to stroke treatment, such as acute kidney injury or cardiac arrest followed by resuscitation, were classified as medical.

In the MM group, the cause of END was classified as ischemia progression (END-IP), cerebral edema, symptomatic hemorrhage, or medical based on follow-up imaging and medical record review according to a previous study (10), with slight modification. Symptomatic hemorrhage was defined as an END due to hemorrhagic lesions with mass effects. Cerebral edema was defined as an END due to edema of initially infarcted tissue with mass effect without hemorrhagic transformation. Ischemia progression was defined as an END due to the expansion of infarction or the development of additional infarctions within the same vascular territory. If there were more than one cause of deterioration, the preceding insult was considered as the cause of END. Medical causes were defined as END due to unexpected, severe medical problems disproportionate to stroke treatment.

### Image Analysis and Occlusion Etiology

Commercial image-viewing software (Picture Archiving and Communication System; Maroview 5.3 Infinitt Co., Seoul, Republic of Korea) was used for image analysis after blinding. The occlusion location was trichotomized to distal basilar, proximal to mid basilar, and vertebral arterial occlusions according to the initiation point of luminal filling defect. The posterior circulation Alberta Stroke Program Early CT score (PC-ASPECTS) was used to grade presenting infarct volume, which was evaluated predominantly on baseline diffusion-weighted images (12). Each assigned point was subtracted when there was more than 20% involvement of the relevant territory (13). Each specific infarct location according to the items on PC-ASPECTS was separately analyzed for its association with END. The Basilar Artery on Computed Tomography Angiography (BATMAN) score (14) and its specific anatomical composition of collateral vessels were utilized to measure baseline collateral status and incorporated into the analyses.

The etiology of VBO was classified as embolic, intracranial-atherosclerotic (15), arterial embolic (16), and dissecting occlusions through review of medical records and serial neuro-images. In the primary EVT group, the etiological classification was sequentially performed according to previous literature (15). For this study, the embolic group was subdivided as arterial embolism if significant culprit stenosis or occlusion was identified proximal to the occlusion of interest (16).

If EVT was not performed/reperfusion not achieved, the occlusion etiology was determined by integrating predictors previously reported, such as the presence of atrial fibrillation, truncal-type occlusions (intracranial atherosclerotic occlusions) vs. branching-site occlusions (embolic occlusions) (17, 18), and severe calcifications (19) at the occlusion site. Serial vessel imaging was also reviewed for recanalization revealing underlying focal stenosis vs. normal lumen (20). Dissections were classified by the identification of intimal flap, double lumen, and intramural hematoma. It was also supplemented by a string of pearls appearance and rapid changes in the vascular morphology.

### Statistical Analysis

In the total VBO population, the patients were classified into the primary EVT group and MM group. Comparative analysis was performed between the two groups. Next, in the primary EVT group, clinical characteristics and pretreatment imaging findings were evaluated for predictors of all-cause END. In the MM population, clinical characteristics and pretreatment imaging findings were evaluated for predictors of END-IP. Next, outcomes of patients that performed rescue EVT due to END were graded. Finally, the patients with inconsistency between institutional EVT inclusion criteria and actual procedure were described. Continuous variables were compared using the Student *t*-test and Mann–Whitney *U*-test, and categorized variables using the chi-square test. Multiple logistic regression was performed for the identification of predictors of END including clinically relevant variables. Data are presented as the mean ± standard deviation, number (%), or median [interquartile range (IQR)] as appropriate. All statistical analyses were performed using IBM SPSS Statistics version 25 (IBM Corp., Armonk, NY, USA). A *p*-value < 0.05 was considered statistically significant.

## Results

### General Characteristics of the Primary EVT Group and MM Group

In total, 174 patients with VBO [age: 67 ± 13, male: 113 (64.9%)] were included in the study, and END occurred in 43 (24.7%) patients. Primary EVT was performed in 66 (37.9%) patients. Occlusion etiology was classified as embolisms in 41 (23.6%), intracranial atherosclerotic occlusions in 108 (62.1%), arterial embolisms in 12 (6.9%), and dissections in 13 (7.5%).

A between-group comparison of patients in the primary EVT group and MM group was performed (Table 1). In clinical parameters, history of hypertension (69.8 vs. 53.0%, *p* = 0.04) and diabetes mellitus (40.6 vs. 24.2%, *p* = 0.043) were more frequently found in the MM group. Stroke onset-to-door time was shorter (2 [1–3] vs. 10 [3–31.5], *p* < 0.001), and initial stroke severity by trichotomized NIHSS groups was higher in the primary EVT group.

**Table 1:** General characteristics of patients in the primary EVT group and MM group.

	**Total (*N* = 174)**	**Primary EVT (*N* = 66)**	**MM (*N* = 108)**	*P*-value^*^
**Clinical parameters**				
Age	67.48 ± 13.03	67.01 ± 13.07	67.78 ± 13.06	0.706
Sex, male	113 (64.9%)	43 (65.2%)	70 (64.8%)	>0.99
Hypertension	109 (63.4%)	35 (53.0%)	74 (69.8%)	0.040
Diabetes mellitus	59 (34.3%)	16 (24.2%)	43 (40.6%)	0.043
Atrial fibrillation	31 (17.8%)	14 (21.2%)	17 (15.7%)	0.477
BMI (kg/m^2^)	24.15 ± 3.42	24.66 ± 3.78	23.84 ± 3.17	0.178
Systolic blood pressure (mmHg)	141.78 ± 20.67	139.21 ± 20.71	143.35 ± 20.59	0.202
Stroke onset-to-door time (h)	4.5 [2–14.75]	2 [1–3]	10 [3–31.5]	<0.001
NIHSS				<0.001
≤ 5	71 (40.8%)	7 (10.6%)	64 (59.3%)	
≤ 20	63 (36.2%)	27 (40.9%)	36 (33.3%)	
>20	40 (23.0%)	32 (48.5%)	8 (7.4%)	
IV thrombolysis	45 (25.9%)	36 (54.6%)	9 (8.3%)	<0.001
**Imaging parameters**				
Occlusion site				0.040
Bilateral VA	65 (37.4%)	17 (25.8%)	48 (44.4%)	
Prox. to mid-basilar	91 (52.3%)	42 (63.6%)	49 (45.4%)	
Distal basilar	18 (10.3%)	7 (10.6%)	11 (10.2%)	
Truncal-type occlusion	115 (66.1%)	31 (47.0%)	84 (77.8%)	<0.001
Incomplete occlusion	43 (24.7%)	7 (10.6%)	36 (33.3%)	0.001
Occlusion etiology				<0.001
Embolism	41 (23.6%)	25 (37.9%)	16 (14.8%)	
Intracranial atherosclerotic	108 (62.1%)	31 (47.0%)	77 (71.3%)	
Arterial embolism	12 (6.9%)	8 (12.1%)	4 (3.7%)	
Dissection	13 (7.5%)	2 (3.0%)	11 (10.2%)	
PC-ASPECTS	8 [8–10]	8 [7–9]	9 [8–10]	<0.001
Thalamic involvement				0.004
Unilateral	17 (9.8%)	8 (12.1%)	9 (8.3%)	
Bilateral	11 (6.3%)	9 (13.6%)	2 (1.9%)	
Cerebellar involvement	53 (30.5%)	22 (33.3%)	31 (28.7%)	0.635
Occipital lobe involvement	18 (10.3%)	10 (15.2%)	8 (7.4%)	0.170
Midbrain involvement	21 (12.1%)	14 (21.2%)	7 (6.5%)	0.008
Pontine involvement	61 (35.1%)	28 (42.4%)	33 (30.6%)	0.153
BATMAN collateral score	6 [4–7.75]	5 [3–6]	6.5 [5–8]	<0.001
P-com subscore	2 [0–3]	1 [0–2]	2 [0–4]	0.067
PCA subscore	2 [1.25–2]	2 [0–2]	2 [2–2]	0.003
Basilar subscore	2 [1–2.75]	1 [1–2]	2 [1.75–3]	<0.001
VA subscore	1 [0–1]	1 [0–1]	1 [0–1]	0.230
Presence of fetal type PCA				0.039
None	126 (72.4%)	55 (83.3%)	71 (65.7%)	
Unilateral	33 (19.0%)	7 (10.6%)	26 (24.1%)	
Bilateral	15 (8.6%)	4 (6.1%)	11 (10.2%)	
**Functional outcomes**				
END	43 (24.7%)	17 (25.8%)	26 (24.1%)	0.945
Δ NIHSS ≥ 4	31 (17.8%)	11 (16.7%)	20 (18.5%)	0.916
3 months mRS	3 [1–5]	5 [2–5]	2 [1–5]	<0.001
Good outcomes	83 (47.7%)	23 (34.9%)	60 (55.6%)	0.013

* *Primary EVT group vs. MM group*.

*EVT, endovascular treatment; MM, medical management; NIHSS, National Institutes of Health Stroke Scale; IV, intravenous; Prox, proximal; VA, vertebral artery; PC-ASPECTS, posterior circulation Alberta Stroke Program Early CT score; BATMAN, The Basilar Artery on Computed Tomography Angiography score; P-com, posterior communicating artery; PCA, posterior cerebral artery; END, early neurological deterioration; eTICI, expanded treatment in cerebral ischemia; mRS, modified Rankin Score*.

Among imaging parameters, more truncal-type occlusions (77.8 vs. 47.0%, *p* < 0.001) and incomplete occlusions (33.3 vs. 10.6%, *p* = 0.001) were seen, with a higher proportion of intracranial atherosclerotic occlusions (71.3 vs. 47.0%, *p* < 0.001) in the MM group. Larger initial infarct volumes represented by PC-ASPECTS (8 [7–9] vs. 9 [8–10], *p* < 0.001), poorer collateral status represented by BATMAN collateral score (5 [3–6] vs. 6.5 [5–8], *p* < 0.001), and absent fetal-type posterior cerebral arteries (PCA) were observed in the primary EVT group.

The rate of ENDs that occurred in the two groups was similar (24.1 vs. 25.8%, *p* = 0.945). Functional outcome, represented by 3 months mRS, was poorer (5 [2–5] vs. 2 [1–5], *p* < 0.001) in the primary EVT group.

### Distribution of END

**Figure 1:**
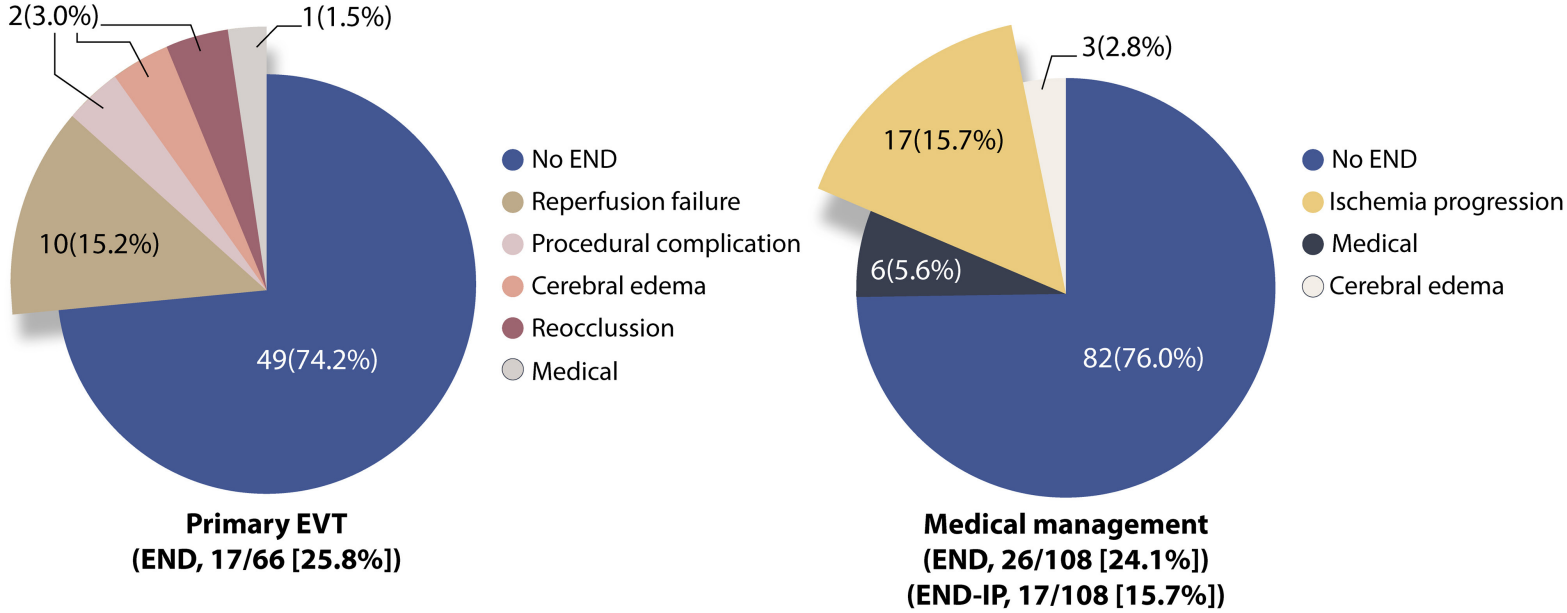
The distribution of causes of early neurological deterioration in the primary endovascular treatment group and medical management group. END, early neurological deterioration; EVT, endovascular treatment; END-IP, early neurological deterioration-ischemia progression.

In the primary EVT group (*N* = 66), END occurred in 17 patients (25.8%). Among them, the causes of END were reperfusion failure in 10 (15.2%), procedure-related in 2 (3.0%), cerebral edema in 2 (3.0%), re-occlusion in 2 (3.0%), and medical in 1 (1.5%). In the MM group (*N* = 108), END occurred in 26 patients (24.1%) and its causes were ischemia progression in 17 (15.7%), cerebral edema in 3 (2.8%), and medical in 6 (5.6%) (Figure 1).

### Predictors of All-Cause END in VBO With Primary EVT

In total, 66 patients were treated with primary EVT. An eTICI ≥ 2b67 was achieved in 40/66 (60.6%) of the patients, and eTICI ≥ 2b50 was achieved in 47/66 (71.2%). END occurred in 17/66 (25.8%) and resulted in ΔNIHSS ≥ 4 in 11/17 (64.7%) at discharge, with a significantly poorer 3-month median mRS compared to the no-END group (5 [4.5–6] vs. 4 [1–5], *p* < 0.001). On comparison of the patients that experienced END and those who did not, there was a frequency of distal basilar occlusions compared to proximal to mid basilar and VA occlusions in the END group (*p* = 0.013) (Table 2). Specific locations of infarction according to the items on PC-ASPECTS and anatomical composition of collaterals by BATMAN collateral scores were not significantly different between the END and no-END groups. The rate of eTICI ≥ 2b67 was lower (35.3 vs. 69.4%, *p* = 0.013) in the END group, while the differences in patients that achieved eTICI ≥ 2b50 did not reach clinical significance.

**Table 2:** Prediction of END in vertebrobasilar artery occlusive stroke patients with primary EVT.

	**Univariate analysis**			**Multivariate analysis**	
	**No-END (*N* = 49)**	**END (*N* = 17)**	***P*-value**	**OR [95% CI]**	***P*-value**
**Clinical parameters**					
Age	67 ± 14	67 ± 12	0.883		
Sex, male	32 (65.3%)	11 (64.7%)	0.964		
Hypertension	28 (57.1%)	7 (41.2%)	0.256		
Diabetes mellitus	11 (22.4%)	5 (29.4%)	0.564		
Atrial fibrillation	11 (22.4%)	3 (17.6%)	0.676		
BMI (kg/m^2^)	24.6 [22.1–26.2]	24.0 [20.1–28.7]	0.908		
Systolic blood pressure (mmHg)	137 ± 21	143 ± 16	0.368		
Stroke onset-to-door time (h)	2 [1.0–3.5]	2 [1.0–3.5]	0.478		
NIHSS			0.170		0.358
≤ 5	5 (10.2%)	2 (11.8%)		Reference	
≤ 20	17 (34.7%)	10 (58.8%)		1.8 [0.2–13.6]	0.568
>20	27 (55.1%)	5 (29.4%)		0.6 [0.1–13.6]	0.654
IV thrombolysis	25 (51.0%)	11 (64.7%)	0.488		
**Imaging parameters**					
Occlusion site			0.013		0.044
Bilateral VA	14 (28.6%)	3 (17.6%)		Reference	
Prox. to mid-basilar	33 (67.3%)	9 (52.9%)		0.6 [0.1–5.1]	0.881
Distal basilar	2 (4.1%)	5 (29.4%)		14.5 [1.4–154.4]	0.027
Truncal-type occlusion	22 (44.9%)	9 (52.9%)	0.567		
Incomplete occlusion	6 (12.2%)	1 (5.9%)	0.463		
Occlusion etiology			0.823		
Embolism	19 (38.8%)	6 (35.3%)			
Intracranial atherosclerotic	22 (44.9%)	9 (52.9%)			
Arterial embolism	6 (12.2%)	2 (11.8%)			
Dissection	2 (12.2%)	0 (0.0%)			
PC-ASPECTS	8 [7–10]	8.0 [5.5–9.0]	0.248		
Thalamic involvement			0.465		
Unilateral	5 (10.2%)	3 (17.6%)			
Bilateral	8 (16.3%)	1 (5.9%)			
Cerebellar involvement	14 (28.6%)	8 (47.1%)	0.233		
Occipital lobe involvement	7 (14.3%)	3 (17.7%)	0.709		
Midbrain involvement	8 (16.3%)	6 (35.3%)	0.165		
Pontine involvement	20 (40.8%)	8 (47.1%)	0.778		
BATMAN collateral score	5 [3–6]	5 [4.0–6.0]	0.085	0.8 [0.6–1.2]	0.249^*^
P-com subscore	1 [0–2]	2 [1–4]	0.257		
PCA subscore	2 [0–2]	1 [0–2]	0.297		
Basilar subscore	1 [1–2]	1 [1–2]	0.347		
VA subscore	1 [0–1]	1 [0–1]	0.827		
Presence of fetal type PCA			0.855		
None	40 (81.6%)	15 (88.2%)			
Unilateral	6 (12.2%)	1 (5.9%)			
Bilateral	3 (6.1%)	1 (5.9%)			
**Procedural parameters**					
Door-to-puncture time (min)	120 [104–148]	131.5 [116.5–178.75]	0.237		
Procedure type			0.083		
Diagnostic/approach failure	6 (12.2%)	6 (35.3%)			
Thrombectomy alone	21 (42.9%)	4 (23.5%)			
Angioplasty/IA tirofiban	22 (44.9%)	7 (41.2%)			
Total procedure time (min)	61 [38–98]	63.5 [35.5–94.25]	0.932		
eTICI ≥ 2b50	38 (77.6%)	9 (52.9%)	0.053		
eTICI ≥ 2b67	34 (69.4%)	6 (35.3%)	0.013	5.0 [1.3–19.9]	0.023^†^
**Functional outcomes**					
Δ NIHSS ≥4	0 (0.0%)	11 (64.7%)	<0.001		
3 months mRS	4 [1.0–5.0]	5 [4.5–6]	<0.001		
Good outcomes	23 (42.6%)	0 (0.0%)	0.005		

* *Per 1 score decrease*.

† *For reperfusion failure*.

*END, early neurological deterioration; OR, odds ratio; CI, confidence interval; BMI, body mass index; NIHSS, National Institutes of Health Stroke Scale; IV, intravenous; VA, vertebral artery; Prox, proximal; PC-ASPECTS, posterior circulation Alberta Stroke Program Early CT score; BATMAN, The Basilar Artery on Computed Tomography Angiography score; P-com, posterior communicating artery; PCA, posterior cerebral artery; IA, intra-arterial; eTICI, expanded treatment in cerebral ischemia; mRS, modified Rankin Score*.

In the multivariable analysis, a distal basilar occlusion location (bilateral VA as reference, OR, 14.5 [95% confidence interval (CI), 1.4–154.4], *p* = 0.027) and failure to achieve eTICI2b67 (OR, 4.9 [95% CI, 1.3–18.7], *p* = 0.019) was associated with END, when trichotomized NIHSS scores and BATMAN collaterals were incorporated as covariates. The occlusion etiology of seven distal basilar occlusion patients was embolic in five, and arterial embolism in two. All showed branching-site occlusion patterns of thrombus involving the basilar bifurcation. Among the five patients with distal basilar occlusion that experienced END, two were due to procedural hemorrhagic complications (subarachnoid hemorrhage and intraventricular hemorrhage), two due to reperfusion failure, and one due to cerebral edema.

### Predictors of END Due to Ischemia Progression in Medically Managed VBO

In the MM group (*N* = 108), END-IP occurred in 17 patients (15.7%). The END-IP group showed higher systolic blood pressure (SBP) at presentation (155 ± 22 vs. 142 ± 20 mmHg, *p* = 0.012), and lower BATMAN collateral scores (5 [4–6.5] vs. 7 [5–9], *p* = 0.030). END-IP was highly associated with poorer mRS at 3 months (1 [1–4] vs. 4 [4–5], *p* < 0.001). When specific infarction locations were analyzed, more thalamic (23.1 vs. 6.1%, *p* = 0.022), cerebellar (46.2 vs. 23.2%, *p* = 0.045), and pontine (50.0 vs. 24.4%, *p* = 0.026) involvements were observed in the END-IP group. Lower posterior communicating artery (P-com) subscores in BATMAN collateral scores (0.5 [0–2] vs. 2 [1–4], *p* = 0.001) were also observed in the END-IP group. The absence of fetal-type PCA was slightly more frequent in the END-IP group (84.6 vs. 59.8%), although it did not reach statistical significance.

In the multivariable analysis, higher SBP at presentation (per 10 mm Hg increase, OR, 1.5 [95% CI, 1.1–2.1], *p* = 0.012), onset-to-door time less than 24 h (OR, 5.3 [95% CI, 1.1–2.0], *p* = 0.049), incomplete occlusions (OR, 4.9 [95% CI, 1.2–19.6], *p* = 0.024), larger infarct cores (per 1 point decrease in PC-ASPECTS, OR, 1.6 [95% CI, 1.0–2.5], *p* = 0.042), and poorer collaterals (per 1 point decrease in BATMAN scores, OR, 1.56 [95% CI, 1.1–2.2], *p* = 0.018) were associated with END-IP, along with trichotomized presenting NIHSS as covariable (Table 3).

**Table 3:** Prediction of END due to ischemia progression in vertebrobasilar artery occlusive stroke patients managed medically.

	**Univariate analysis**			**Multivariate analysis**	
	**No-END-IP (*N* = 91)**	**END-IP (*N* = 17)**	***P*-value**	**OR [95% CI]**	***P*-value**
**Clinical parameters**					
Age (years)	68 ± 13	65 ± 14	0.277		
Sex, male	59 (64.8%)	11 (64.7%)	0.992		
Hypertension	63 (69.2%)	11 (64.7%)	0.728		
Diabetes mellitus	34 (37.4%)	9 (52.9%)	0.430		
Atrial fibrillation	16 (17.6%)	1 (5.9%)	0.224		
BMI (kg/m^2^)	23.9 [24.1–25.7]	24.3 [22.5–26.9]	0.368		
Systolic blood pressure (mmHg)	142 ± 20	155 ± 22	0.018	1.5 [1.1–2.0]	0.012^*^
Stroke onset-to-door time	10 [3–37]	8 [3–22.5]	0.383		
Stroke onset-to-door time			0.394		0.049
<24 h	60 (65.9%)	13 (76.5%)		5.3 [1.1–2.0]	
≥24 h	31 (34.1%)	4 (23.5%)		Reference	
Trichotomized NIHSS			0.115		0.994
≤ 5	56 (61.5%)	8 (47.1%)			
≤ 20	27 (29.7%)	9 (52.9%)			
>20	8 (8.8%)	0 (0.0%)			
IV thrombolysis	5 (5.5%)	4 (23.5%)	0.033		
**Imaging parameters**					
Occlusion site			0.460		
Distal basilar	10 (11.0%)	1 (5.9%)			
Prox. to mid-basilar	39 (42.9%)	10 (58.8%)			
Bilateral VA	42 (46.2%)	6 (35.3%)			
Truncal-type occlusion	69 (75.8%)	15 (88.2%)	0.259		
Incomplete occlusion	27 (29.7%)	9 (52.9%)	0.062	4.9 [1.2–19.6]	0.024
Occlusion etiology			0.801		
Embolism	14 (15.4%)	2 (11.8%)			
Intracranial atherosclerotic	64 (70.3%)	13 (76.5%)			
Arterial embolism	4 (4.4%)	0 (0.0%)			
Dissection	9 (9.9%)	2 (11.8%)			
PC-ASPECTS	10 [8–10]	8 [7–9.5]	0.059	1.6 [1.0–2.5]	0.042^†^
Thalamic involvement			0.027		
Unilateral	4 (4.9%)	5 (19.2%)			
Bilateral	1 (1.2%)	1 (3.9%)			
Cerebellar involvement	19 (23.2%)	12 (46.2%)	0.045		
Occipital involvement	6 (7.3%)	2 (7.7%)	>0.99		
Midbrain involvement	3 (3.7%)	4 (15.4%)	0.056		
Pontine involvement	20 (24.4%)	13 (50.0%)	0.026		
BATMAN collateral score	7 [5–9]	5 [4–6.5]	0.030	1.6 [1.1–2.2]	0.018^†^
P-com subscore	2 [1–4]	0.5 [0–2]	0.001		
PCA subscore	2 [2–2]	2 [1–2]	0.051		
Basilar subscore	2 [2–3]	2 [1–3]	0.479		
VA subscore	1 [0–1]	1 [0–1]	0.455		
Presence of fetal type PCA			0.077		
None	49 (59.8%)	22 (84.6%)			
Unilateral	23 (28.1%)	3 (11.5%)			
Bilateral	10 (12.2%)	1 (3.9%)			
**Functional outcomes**					
3 months mRS	1 [1–4]	4 [4–5]	<0.001		
Good outcome	58 (63.7%)	2 (11.8%)	<0.001		

* *Per 10 mmHg increase*.

† *Per 1 score decrease*.

*END, early neurological deterioration; END-IP, END due to ischemia progression; OR, odds ratio; CI, confidence interval; NIHSS, National Institutes of Health Stroke Scale; IV, intravenous; VA, vertebral artery; Prox, proximal; PC-ASPECTS, posterior circulation Alberta Stroke Program Early CT score; BATMAN, The Basilar Artery on Computed Tomography Angiography score; P-com, posterior communicating artery; PCA, posterior cerebral artery; mRS, modified Rankin Score*.

### Rescue EVT

Rescue EVTs were performed in seven patients after the occurrence of END during the admission course. Six patients were from the MM group, and one patient was from the primary EVT group. Among them, six of seven (85.7%) occlusions were presumed to be atherosclerotic in origin and required additional EVTs such as angioplasty and/or intra-arterial tirofiban, other than first-line mechanical thrombectomy. Even though eTICI ≥ 2b67 reperfusion was achieved in five of seven (71.5%), no patients achieved functional independence at 3 months, while 42.9% were bedridden or had died (Table 4).

**Table 4:** Clinical characteristics and outcome of patients who underwent rescue EVT therapy.

**No**	**Sex/age**	**O-to-D time (h)**	**NIHSS**	**Occlusion type**	**Infarct location**	**Occlusion etiology**	**PC-ASPECTS**	**BATMAN**	**Cause of END and imaging findings**	**Onset to rescue EVT**	**Procedure type**	**eTICI**	**3-m mRS**
**Primary EVT group**													
1	M/55	1	6	BSO	Bilateral cerebellum, thalamus	Arterial embolism	4	4	Reocclusion: arterial re-embolism	7 hours	Thrombectomy/ angioplasty	3	6
**Medical management group**													
2	M/72	16	4	TTO	Bilateral pons, Rt cerebellum	ICAS	7	9	END-IP, near-total occlusion to complete occlusion	4 days	Thrombectomy	2b67	6
3	M/59	1	7	TTO	Rt pons	ICAS	8	6	END-IP, near-total occlusion to complete occlusion	3 days	Angioplasty/ IA tirofiban	2a	6
4	F/81	3	0	TTO	Bilateral pons	ICAS	10	7	END-IP, no vessel change	5 days	IA tirofiban	2b50	4
5	F/76	11	4	TTO	Lt pons	ICAS	8	7	END-IP, near-total occlusion to complete occlusion	1 day	IA tirofiban	2b67	4
6	F/73	8	8	TTO	Rt cerebellum, Bilateral occipital	ICAS	9	5	END-IP, no vessel change	2 days	IA tirofiban	2b67	4
7	F/80	5	9	TTO	Rt pons	ICAS	8	2	END-IP, elongation of near-total occlusion length	1 day	Thrombectomy/ IA tirofiban	3	4

*No, number; O-to-D, onset-to-door; NIHSS, National Institutes of Health Stroke Scale; PC-ASPECTS, posterior circulation Alberta Stroke Program Early CT score; BATMAN, The Basilar Artery on Computed Tomography Angiography score; END, early neurological deterioration; EVT, endovascular therapy; eTICI, expanded treatment in cerebral ischemia; mRS, modified Rankin Score, M, male; F, female; BSO, branching-site occlusion, TTO; truncal-type occlusion; Rt, right; Lt, left; ICAS; intracranial atherosclerosis; END-IP, END due to ischemia progression; IA, intra-arterial*.

### Deviation Between Institutional EVT Criteria and Actual Implementation

Overall, five patients did not meet the institutional inclusion criteria for EVT due to low NIHSS, but received EVT (Table 5). In three (60.0%) patients, basilar top occlusions were observed which the clinician considered as a sign predictive of END, while in two (40.0%) other patients, younger age of onset led to a more aggressive treatment decision.

**Table 5:** Brief description of patients who did not meet the institutional inclusion criteria for EVT, however did receive the EVT.

**No**	**Year**	**Sex/** **age**	**O-to-d time (h)**	**NIHSS**	**IV tPA**	**Infarct location**	**Occlusion etiology**	**PC-ASPECTS**	**BAT-MAN**	**Reason for deviation**	**END and cause**	**Procedure type**	**eTICI**	**3-m mRS**
1	2011	M/40	3	3	Yes	Rt lat medullary, PICA scattered infarct	Arterial embolism	10	4	Basilar top occlusion		Diagnostic	2a	2
2	2012	M/74	1	4	Yes	Lt portion of the corpus callosum body	Embolism	10	5	Basilar top occlusion		Thrombectomy	3	0
3	2014	F/71	5	2	-	Lt paramedian pontine & Lt PCA scattered	Embolism	10	6	Basilar top occlusion		Diagnostic	0	0
4	2013	M/57	4	2	Yes	Lt cerebellar	Dissection	8	4	Young agemidbasilar occlusion	Reperfusion failure	Thrombectomy, angioplasty, IA tirofiban	2b50	5
5	2013	M/61	1	4	Yes	Lt paramedian pons	ICAS	7	6	Young ageneurological fluctuation		Thrombectomy, angioplasty	2b67	0

*No, number; O-to-D, onset-to-door; NIHSS, National Institutes of Health Stroke Scale; IV tPA, intravenous tissue plasminogen activator; PC-ASPECTS, posterior circulation Alberta Stroke Program Early CT score; BATMAN, The Basilar Artery on Computed Tomography Angiography score; END, early neurological deterioration; EVT, endovascular therapy; eTICI, expanded treatment in cerebral ischemia; mRS, modified Rankin Score, M, male; F, female; Rt, right; Lt, left; PCA, posterior cerebral artery; PICA, posterior inferior cerebellar artery; ICAS; intracranial atherosclerosis; IA, intra-arterial*.

On the contrary, EVT was not performed on 21 patients despite fulfilling the clinical severity and time criteria (Table 6). Among them, four were socioeconomic decisions or related to poor baseline functional status. In the rest of the 17 patients, six (35.3%) were because the clinician had doubts about the benefits of EVT in obviously chronic occlusions. In four (23.5%) patients, suspicion for dissecting occlusion led to the deviation. In another four (23.5%) patients, there were clinical improvements before EVT.

**Table 6:** Brief description of patients who met the institutional indication for EVT decision, however did not receive EVT.

**No**	**Year**	**Sex/age**	**O-to-d time (h)**	**NIHSS**	**IV tPA**	**Infarct location**	**Occlusion etiology**	**PC-ASPECTS**	**BAT-MAN**	**Reason for deviation**	**END and cause**	**3-m mRS**
**EVT not performed based on clinical factors**												
1	2011	F/74	5	12	-	Bilateral pontine	ICAS	6	6	Chronic occlusion, long prodrome		4
2	2012	F/78	3	7	-	Bilateral paramedian pontine	ICAS	8	9	Chronic occlusionClinically lacunar syndrome		4
3	2014	M/80	1	15	-	Rt pontine, Lt cerebellar, Lt MCA scattered	ICAS	8	3	Chronic occlusion, long prodrome	Medical	5
4	2015	M/72	1	9	-	Bilateral pontine, Rt lateral medullary	ICAS	8	9	Chronic occlusion, incomplete occlusion		5
5	2016	F/68	2	5	-	Rt medial medullary	ICAS	10	6	Chronic occlusionStroke not due to perfusion failure		3
6	2017	M/64	1	14	-	Bilateral medial medullary, Lt cerebellar scattered	ICAS	10	5	Chronic occlusion, incomplete occlusion		2
7	2015	M/41	3	6	Yes	Rt lateral medullary	Dissection	10	10	Cannot rule out dissectionStroke not due to perfusion failure		1
8	2013	M/30	2	7	Yes	Bilateral paramedian pontine	Dissection	8	9	Cannot rule out dissection	END-IP	5
9	2014	M/55	0	19	-	Bilateral pontine	ICAS	10	9	Cannot rule out dissection		3
10	2014	M/54	1	8	Yes	Lt PICA, Rt posterior choroidal, Rt SCA scattered	Arterial embolism	9	8	Cannot rule out dissection		3
11	2012	M/62	2	16	Yes	Lt pontine	Embolism	10	6	Improving neurological state	END-IP	4
12	2013	M/72	1	13	-	Lt occipital	ICAS	10	9	Improving neurological state		5
13	2017	M/76	5	10	-	Bilateral pontine and cerebellar scattered	ICAS	8	7	Improving neurological state	Medical	5
14	2018	F/89	3	10	-	Rt hippocampal	Embolism	10	4	Improving neurological state		2
15	2011	M/67	3	18	-	Bilateral cerebellar scattered	Arterial embolism	7	7	Incomplete occlusion		3
16	2011	M/71	1	31	-	Bilateral pontine and cerebellar	Arterial embolism	6	5	Large infarct core, in-hospital delay		6
17	2011	F/82	4	19	-	Bilateral thalamus	Embolism	10	4	Cardiogenic shock		5
**EVT not performed based on socioeconomic factors**												
18	2011	F/86	1	30	Yes	Lt thalamus and cerebellar	Embolism	8	3	Caregiver refusal	Medical	5
19	2013	F/99	1	18	Yes	Lt PCA, Rt pontine, Rt cerebellar	Embolism	5	3	Caregiver refusal	Cerebral edema	5
20	2017	M/81	2	15	-	Lt PICA territory, Lt pontine, bilateral PCA territory	ICAS	7	3	Caregiver refusal	Medical	6
21	2018	F/91	1	31	-	Bilateral pontine, Lt AICA	Embolism	5	2	Caregiver refusal		6

*No, number; O-to-D, onset-to-door; NIHSS, National Institutes of Health Stroke Scale; IV tPA, intravenous tissue plasminogen activator; PC-ASPECTS, posterior circulation Alberta Stroke Program Early CT score; BATMAN, The Basilar Artery on Computed Tomography Angiography score; END, early neurological deterioration; EVT, endovascular therapy; eTICI, expanded treatment in cerebral ischemia; mRS, modified Rankin Score, M, male; F, female; Rt, right; Lt, left; MCA, middle cerebral artery; SCA, superior cerebellar artery; PCA, posterior cerebral artery; PICA, posterior inferior cerebellar artery; AICA, anterior inferior cerebellar artery; ICAS; intracranial atherosclerosis; END-IP, END due to ischemia progression*.

## Discussion

This single-center retrospective study explores the potential predictors of END in stroke due to VBO, in regard to the different treatment populations, which are the primary EVT group and MM group. In this study, we found that in the primary EVT group, failure to achieve eTICI2b67 reperfusion and distal basilar occlusion were associated with all-cause END. In the MM group, higher SBP at presentation, onset-to-door time less than 24 h, incomplete occlusions, larger infarct cores, and poorer collaterals were associated with END-IP. There were also deviations between institutional EVT criteria (based on time and clinical severity) and actual implementation. In this study, the clinicians' decisions were biased toward implementation of EVT in patients with basilar top occlusions, and against EVT in patients with suspicious dissecting occlusions or chronic intracranial atherosclerotic occlusions.

A factor predictive of END in the primary EVT group was failure to achieve reperfusion of more than 66% of the involved territory (eTICI2b67). In contrast, more classical reperfusion of more than 50% of the involved territory (mTICI2b) failed to predict END. Compared to the anterior circulation, EVT for posterior circulation is prone to Willisian collateral failure by distal embolization (21). Thus, aiming for a higher reperfusion grade may be able to prevent END on VBO, and future advances in EVT may also need to focus on higher reperfusion grades, such as distal vessel thrombectomy (22). A selective reperfusion grading system for VBO may also maximize EVT treatment effect.

Distal basilar occlusions were also associated with END. The causes of END were reperfusion failure and procedural complications. A distal basilar occlusion is likely to be embolic, with the main thrombus burden in the basilar bifurcation, or the posterior cerebral artery. Considering this, technical difficulty may have been the reason for the association between END and distal basilar occlusions. For example, a saddle-shaped thrombus at the arterial bifurcation point may be more resistant to stent retrieval thrombectomy (23). Extended manipulation may also injure the perforators at the basilar tip. The only two cases of procedure-related hemorrhagic complications in this study occurred in this group. Hence, neuro-interventionalists should be wary of procedural complications to prevent END in distal basilar occlusions, and utilizing contact aspiration (24) or dual-stent retrievers (23) as the first-line thrombectomy method may be useful.

Previously known factors associated with the prognosis of VBO were also analyzed for its relation to END in our study. For example, factors such as bilateral thalamic involvement (25), presence of P-com collaterals (26), infarct burden (12), or total collaterals (14) were previously reported. In our study, within the MM group, total infarct burden and collaterals could predict END. In specific, lower P-com collateral subscores were associated with END, while a fetal type PCA tended to be reversely associated with END, but did not reach clinical significance. The protective effect of presence and caliber of P-com collaterals have been consistently reported (26, 27), while for fetal-type PCA, there are contradicting results, which are likely affected by recanalization or stroke mechanisms. In terms of infarct location and burden, lesions in the bilateral thalamus, pons, and cerebellum may be associated with critical perfusion failure of the posterior circulation; future research is warranted. Interestingly, such associations were not seen in the primary EVT group, suggesting that END after EVT may be largely dependent on the consequence of reperfusion.

While a wide definition of END (1) and differences between anterior and posterior circulation (7) bring confusion to the clinical significance of a given END definition, our results demonstrate that END, classified as an increase of 4 or more in the NIHSS score, during medical management is a critical event. Moreover, a large percentage of END was due to ischemia progression, while END due to symptomatic hemorrhage was less common compared with the anterior circulation (10, 28). In a previous study, we have evaluated predictors of neurological deterioration, classified as an increase in NIHSS of 4 or more at discharge, in a smaller number of patients with medically managed VBO (29). Even though the current study differs from the previous in that END was described as an increase in NIHSS of 4 or more during admission, and selectively identified END due to ischemia progression, the important predictors of END due to ischemia progression did not differ largely.

The outcomes of rescue EVT were unsatisfactory, showing median 3 months mRS of 5. The majority were due to intracranial atherosclerotic occlusions, and a likely explanation is that underlying intracranial atherosclerotic disease can cause END by various mechanisms (30). If the main mechanism is due to *in situ* occlusion and hemodynamic impairment, rescue EVT may result in neurological improvement, but if the main mechanism is due to arterial embolism or local branch occlusion, neurological improvement would be less likely. Considering the poor outcomes associated with END and unsatisfactory results of rescue EVT, a high-risk group for END in patients with VBO may benefit from preemptive EVT. This is supported by recent literature advocating for primary EVT in patients with anterior circulation large-vessel occlusions and low NIHSS scores (31). Considering a predominance of intracranial atherosclerotic occlusions in this group, a rational EVT plan would be to achieve luminal flow by atraumatic stent-retrieval thrombectomy, followed by intra-arterial antiplatelet agent injections to stabilize the endothelium (19).

There were deviations from institutional EVT criteria and actual implementation. While this may be a potential source of bias for the study analysis, it can also be valuable data showing that clinicians' concerns for END influences EVT decision. VBO with the involvement of the basilar top led the clinician to anticipate END without definitive reperfusion measures, which is justifiable considering the critical role of the basilar top in the Willisian collaterals. In such cases, manual aspiration thrombectomy may be chosen over stent retriever thrombectomy (24) to prevent END associated with vessel trauma. On the contrary, suspicion for dissecting occlusions led the clinician to worry about END associated with endovascular procedures. Subarachnoid hemorrhage due to vessel rupture during microcatheter navigation (32) and inadvertent stenting of pseudolumen are feared complications. Direct aspiration may be chosen first to theoretically avoid such complications (32). Chronic VBO may also deteriorate with EVT due to its reduced arterial caliber or difficulties in the lesion approach. However, when considering that rescue EVT outcomes were unsatisfactory, future studies are warranted regarding optimal treatment time and method in this population. While current evidence is limited, a recent literature regarding EVT for non-acute intracranial vertebral arterial occlusions reports acceptable outcomes with careful classification (33).

Several limitations exist for this study. First, the retrospective design and imaging-based inclusion criteria may include patients with heterogeneous stroke mechanisms, and mixed acute and chronic VBO. As our study goal was to find clues for maximizing EVT effect and expanding the pool of patients that will benefit from EVT in VBO, this heterogeneity was intended. However, this study was performed in a population with a higher proportion of intracranial atherosclerosis, and implementation of its results may vary according to ethnic differences. Second, in the primary EVT group, a more detailed analysis of collateral changes or distal embolizations and their consequences could not be performed. We hope to address such issues in a larger number of VBO patients undergoing reperfusion. Third, while we show predictors of END in VBO managed medically, and poor outcomes of rescue EVT, we could not provide evidence that a preemptive EVT may improve patient outcomes. Future prospective studies will be needed to address this issue. Fourth, while all EVT patients were primarily treated with stent retriever thrombectomy or direct aspiration thrombectomy, there may be heterogeneity in EVT methods due to the rather long period of patient inclusion. The following may have influenced the EVT methods; in Korea, the Penumbra system was approved in November 2008 and reimbursed in August 2009 in Korea. The second-generation Penumbra system was approved in July 2014. Solitaire AB has been approved for stent-assisted coiling since May 2010 and was reimbursed in January 2011. The Solitaire FR was approved for mechanical thrombectomy in April 2013 and reimbursed in August 2014. Trevo was approved for mechanical thrombectomy in December 2012 and reimbursed in August 2014 (34).

In conclusion, in patients with stroke due to VBO, several potential predictors of END can be identified according to the different treatment statuses. In the primary EVT group, failure to achieve reperfusion and distal basilar occlusion were associated with all-cause END. In the MM group, higher SBP at presentation, onset-to-door time less than 24 h, incomplete occlusions, larger infarct cores, and poorer collaterals were associated with END-IP. Stroke neurologists considered basilar top occlusions to be liable to deteriorate without EVT while dissecting occlusions or chronic intracranial atherosclerotic occlusions to be associated with END related to EVT. Recognization of such variables and addressing these issues may improve overall patient selection and treatment outcomes in VBO.

## Data Availability Statement

The raw data supporting the conclusions of this article will be made available by the authors, without undue reservation.

## Ethics Statement

The studies involving human participants were reviewed and approved by Ajou University Hospital Institutional review Board. Written informed consent for participation was not required for this study in accordance with the national legislation and the institutional requirements.

## Author Contributions

SK: data interpretation, drafted the work, and approved the final version of the paper. SL, WJ, JC, JL, and JH: data interpretation, revised the draft critically for important intellectual content, and approved the final version of the paper. S-JL: conceptualization and supervision of the study, data interpretation, revised the draft critically for important intellectual content, and approved the final version of the paper. All authors have seen and approved the manuscript being submitted.

## Funding

This work was supported by the new faculty research fund of Ajou University School of Medicine (M-2020-C0460-00067).

## Conflict of Interest

The authors declare that the research was conducted in the absence of any commercial or financial relationships that could be construed as a potential conflict of interest. The Handling Editor declared a past co-authorship with two of the authors, JL and JH.

## Publisher's Note

All claims expressed in this article are solely those of the authors and do not necessarily represent those of their affiliated organizations, or those of the publisher, the editors and the reviewers. Any product that may be evaluated in this article, or claim that may be made by its manufacturer, is not guaranteed or endorsed by the publisher.

## References

[1] Siegler JE Martin-Schild S. Early neurological deterioration (END) after stroke: the END depends on the definition. Int J Stroke. (2011) 6:211–12. 10.1111/j.1747-4949.2011.00596.x 21557807

[2] Seners P Ben Hassen W Lapergue B Arquizan C Heldner MR Henon H. Prediction of early neurological deterioration in individuals with minor stroke and large vessel occlusion intended for intravenous thrombolysis alone. JAMA Neurol. (2021) 78:321–8. 10.1001/jamaneurol.2021.2025 33427887 PMC7802007

[3] Seners P Turc G Tisserand M Legrand L Labeyrie MA Calvet D. Unexplained early neurological deterioration after intravenous thrombolysis: incidence, predictors, associated factors. Stroke. (2014) 45:2004–9. 10.1161/STROKEAHA.114.005426 24876087

[4] Girot JB Richard S Gariel F Sibon I Labreuche J Kyheng M. Predictors of unexplained early neurological deterioration after endovascular treatment for acute ischemic stroke. Stroke. (2020) 51:2943–50. 10.1161/STROKEAHA.120.029494 32921260

[5] Langezaal LCM van der Hoeven E Mont'Alverne FJA de Carvalho JJF Lima FO Dippel DWJ. Endovascular therapy for stroke due to basilar-artery occlusion. N Engl J Med. (2021) 384:1910–20. 10.1056/NEJMoa2030297 34010530

[6] Lee SJ Hong JM Choi JW Park JH Park B Kang DH. Predicting endovascular treatment outcomes in acute vertebrobasilar artery occlusion: a model to aid patient selection from the ASIAN KR registry. Radiology. (2020) 294:628–37. 10.1148/radiol.2020191227 31990269

[7] Schneck MJ. Current stroke scales may be partly responsible for worse outcomes in posterior circulation stroke. Stroke. (2018) 49:2565–6. 10.1161/STROKEAHA.118.023201 30355229

[8] Mattle HP Arnold M Lindsberg PJ Schonewille WJ Schroth G. Basilar artery occlusion. Lancet Neurol. (2011) 10:1002–14. 10.1016/S1474-4422(11)70229-0 22014435

[9] Nogueira RG Ribo M. Endovascular treatment of acute stroke. Stroke. (2019) 50:2612–8. 10.1161/STROKEAHA.119.023811 31340728

[10] Kim JM Moon J Ahn SW Shin HW Jung KH Park KY. The etiologies of early neurological deterioration after thrombolysis and risk factors of ischemia progression. J Stroke Cerebrovasc Dis. (2016) 25:383–8. 10.1016/j.jstrokecerebrovasdis.2015.10.010 26597264

[11] Liebeskind DS Bracard S Guillemin F Jahan R Jovin TG Majoie CB. eTICI reperfusion: defining success in endovascular stroke therapy. J Neurointerv Surg. (2019) 11:433–8. 10.1136/neurintsurg-2018-014127 30194109

[12] Puetz V Khomenko A Hill MD Dzialowski I Michel P Weimar C. Extent of hypoattenuation on CT angiography source images in basilar artery occlusion: prognostic value in the Basilar Artery International Cooperation Study. Stroke. (2011) 42:3454–9. 10.1161/STROKEAHA.111.622175 21960577

[13] Khatibi K Nour M Tateshima S Jahan R Duckwiler G Saver J. Posterior circulation thrombectomy-pc-aspect score applied to preintervention magnetic resonance imaging can accurately predict functional outcome. World Neurosurg. (2019) 129:e566–71. 10.1016/j.wneu.2019.05.217 31158539

[14] Alemseged F Shah DG Diomedi M Sallustio F Bivard A Sharma G. The basilar artery on computed tomography angiography prognostic score for basilar artery occlusion. Stroke. (2017) 48:631–7. 10.1161/STROKEAHA.116.015492 28228577

[15] Kim YW Hong JM Park DG Choi JW Kang DH Kim YS. Effect of intracranial atherosclerotic disease on endovascular treatment for patients with acute vertebrobasilar occlusion. AJNR Am J Neuroradiol. (2016) 37:2072–8. 10.3174/ajnr.A4844 27313131 PMC7963784

[16] Baik SH Park HJ Kim JH Jang CK Kim BM Kim DJ. Mechanical thrombectomy in subtypes of basilar artery occlusion: relationship to recanalization rate and clinical outcome. Radiology. (2019) 291:730–7. 10.1148/radiol.2019181924 30912720

[17] Baek JH Kim BM Kim DJ Heo JH Nam HS Song D. Importance of truncal-type occlusion in stentriever-based thrombectomy for acute stroke. Neurology. (2016) 87:1542–50. 10.1212/WNL.0000000000003202 27629085

[18] Baek JH Kim BM Yoo J Nam HS Kim YD Kim DJ. Predictive value of computed tomography angiography-determined occlusion type in stent retriever thrombectomy. Stroke. (2017) 48:2746–52. 10.1161/STROKEAHA.117.018096 28864601

[19] Lee JS Hong JM Kim JS. Diagnostic and therapeutic strategies for acute intracranial atherosclerosis-related occlusions. J Stroke. (2017) 19:143–51. 10.5853/jos.2017.00626 28592778 PMC5466291

[20] Lee JS Lee SJ Yoo JS Hong JH Kim CH Kim YW. Prognosis of acute intracranial atherosclerosis-related occlusion after endovascular treatment. J Stroke. (2018) 20:394–403. 10.5853/jos.2018.01627 30309234 PMC6186924

[21] Yeo LLL Holmberg A Mpotsaris A Soderman M Holmin S Kuntze Soderqvist A. Posterior circulation occlusions may be associated with distal emboli during thrombectomy: factors for distal embolization and a review of the literature. Clin Neuroradiol. (2019) 29:425–33. 10.1007/s00062-018-0679-z 29569010 PMC6710331

[22] Saver JL Chapot R Agid R Hassan A Jadhav AP Liebeskind DS. Thrombectomy for distal, medium vessel occlusions: a consensus statement on present knowledge and promising directions. Stroke. (2020) 51:2872–84. 10.1161/STROKEAHA.120.028956 32757757

[23] Asadi H Brennan P Martin A Looby S O'Hare A Thornton J. Double stent-retriever technique in endovascular treatment of middle cerebral artery saddle embolus. J Stroke Cerebrovasc Dis. (2016) 25:e9–11. 10.1016/j.jstrokecerebrovasdis.2015.10.005 26698640

[24] Choi JW Han M Park JH Jung WS. Effect of manual aspiration thrombectomy using large-bore aspiration catheter for acute basilar artery occlusion: comparison with a stent retriever system. BMC Neurol. (2020) 20:434. 10.1186/s12883-020-02013-7 33250061 PMC7702718

[25] Yoon W Kim SK Heo TW Baek BH Lee YY Kang HK. Predictors of good outcome after stent-retriever thrombectomy in acute basilar artery occlusion. Stroke. (2015) 46:2972–5. 10.1161/STROKEAHA.115.010840 26330448

[26] Maus V Kalkan A Kabbasch C Abdullayev N Stetefeld H Barnikol UB. Mechanical thrombectomy in basilar artery occlusion: presence of bilateral posterior communicating arteries is a predictor of favorable clinical outcome. Clin Neuroradiol. (2019) 29:153–60. 10.1007/s00062-017-0651-3 29260256

[27] van der Hoeven EJ McVerry F Vos JA Algra A Puetz V Kappelle LJ. Collateral flow predicts outcome after basilar artery occlusion: the posterior circulation collateral score. Int J Stroke. (2016) 11:768–75. 10.1177/1747493016641951 27016515

[28] Valentino F Gentile L Terruso V Mastrilli S Aridon P Ragonese P. Frequency and determinants for hemorrhagic transformation of posterior cerebral stroke: posterior ischemic stroke and hemorrhagic transformation. BMC Res Notes. (2017) 10:592. 10.1186/s13104-017-2889-x 29132407 PMC5683579

[29] Koh S Park JH Park B Choi MH Lee SE Lee JS. Prediction of infarct growth and neurological deterioration in patients with vertebrobasilar artery occlusions. J Clin Med. (2020) 9:3759. 10.3390/jcm9113759 33266388 PMC7700123

[30] Kim JS Nah HW Park SM Kim SK Cho KH Lee J. Risk factors and stroke mechanisms in atherosclerotic stroke: intracranial compared with extracranial and anterior compared with posterior circulation disease. Stroke. (2012) 43:3313–8. 10.1161/STROKEAHA.112.658500 23160885

[31] Heldner MR Chaloulos-Iakovidis P Panos L Volbers B Kaesmacher J Dobrocky T. Outcome of patients with large vessel occlusion in the anterior circulation and low NIHSS score. J Neurol. (2020) 267:1651–62. 10.1007/s00415-020-09744-0 32062782

[32] Park KH Kwak HS Park JS. Endovascular approach in patients with acute complete occlusion due to middle cerebral artery dissection. J Korean Neurosurg Soc. (2020) 63:717–22. 10.3340/jkns.2020.0053 33105537 PMC7671787

[33] Gao F Sun X Zhang H Ma N Mo D Miao Z. Endovascular recanalization for nonacute intracranial vertebral artery occlusion according to a new classification. Stroke. (2020) 51:3340–3. 10.1161/STROKEAHA.120.030440 32838672

[34] Lee JS Lee SJ Hong JM Choi JW Hong JH Chang HW. Temporal changes in care processes and outcomes for endovascular treatment of acute ischemic stroke: retrospective registry data from three Korean centers. Neurointervention. (2018) 13:2–12. 10.5469/neuroint.2018.13.1.2 29535893 PMC5847886

